# Synthesis of nano-sized lead sulfide thin films from Avocado (*Glycosmis cochinchinensis*) Leaf extracts to empower pollution remediation

**DOI:** 10.1038/s41598-022-15785-4

**Published:** 2022-07-09

**Authors:** Abel Saka, Lamessa Gudata, Leta Tesfaye Jule, Venkatesh Seeivasan, Nagaprasad N, Krishnaraj Ramaswamy

**Affiliations:** 1 Department of Physics, College of Natural and Computational Science, Dambi Dollo University, Dembi Dolo, Ethiopia; 2 Centre for Excellence in Technology Transfer and Incubation, Dambi Dollo University, Dembi Dolo, Ethiopia; 3 Department of Mechanical Engineering, Sri Eshwar College of Engineering, Coimbatore, India; 4 Department of Mechanical Engineering, ULTRA College of Engineering and Technology, Madurai, Tamilnadu 625 104 India; 5 Department of Mechanical Engineering, College of Engineering Science, Dambi Dollo University, Dembi Dolo, Ethiopia

**Keywords:** Biochemistry, Biophysics, Biotechnology, Plant sciences, Climate sciences, Environmental sciences, Environmental social sciences, Chemistry, Energy science and technology, Engineering, Materials science, Nanoscience and technology, Physics

## Abstract

The translucent and nano-crystalline PbS films were equipped with the CBD techniques on metal substrates by the temperature of 90 °C through aqueous solutions of Lead Nitrate and Thiourea. The XRD phases verify the crystalline property of synthesized thin films that the shape falls in the cubic structures with favourite orientations. It revealed that the prepared material is cubic crystal oriented as (111), (110), (100) and (101) crystal planes. The crystalline size varied between 0.4 and 0.7 nm. The band gap was assessed using UV–vis captivation spectra and Tau relations. The average energy band gap was found to be 2.43 eV which is greater than bulk materials of PbS; because of quantum confinements of Lead Sulfide Nano Crystalline thin films, and PL also confirms this result. The variation in band gap with Leaf extracts and particle sizes displayed blue shifts characteristic of electrons quantum confinements. SEM micrograph shows extremely uniform and adherent PbS films are found at higher PH values. It was evidently observed that the viscosity of the synthesized thin films reduced from 563 to 111 nm with a rise in pH value. The sample prepared at pH 4 shows good performance, and thin films deposited from Avocado (*Glycosmis cochinchinensis*) leaf extracts are a promising method to empower pollution remediation and future energy.

## Introduction

Nanomaterials have been acknowledged to have a wide-ranging range of presentations in various areas. Because of their hopeful physicals, optical property and gas detecting behaviours, polymer composite has more and more applicability. Several investigators have studied the preparation of different chalcogenides. The electro-luminescence, as well as numerous diffractive properties of Nano-composites of chalcogenides/polymer, has lately been explored^1–3^.

Nanotechnologies also have an attention-grabbing character in the fields of subtracting, power inducing, optoelectronics, drug deliveries and ecological remediation^4^. In the initiation of Nano-technology, various nano-scale expedients have been advanced using several techniques, such as physicals, chemicals as well as green methods. Until now, a green Nanoparticle deposition is a tool of optimal easily equipped and contrived^5–7^. It is possible to see disadvantages of conservative methods for the production of Nanoparticles, comprising long term treating, expensive, painstaking processes, and in specific the usage of the poisonous compound. Furthermost of the applicable investigations have been engaged in biodegradable and fast production procedures for the manufacture of Nanoparticles because of these limitations^8,9^. The growth of eco-friendly techniques for manufacturing nano-scale ingredients has been the foremost attention in current centuries. In this esteem, green bottle production of Nanoparticles, particularly through leaf extracts from various floras, is increasing development that is considered simpler, cheaper as well as non-toxic in green harmony^10^. Nanotechnology has improved with a humanoid standard of existing through addressing numerous daily life issues, such as the involvement to power supply; weather alteration; gorgeousness, fabric as well as health manufacturing comprising the treatment of fatal sicknesses such as cancer as well as Alzheimer^11–13^. Because of their various claims in numerous procedural areas, inclusive study into metallic sulfide Nanoparticles has been concerted in previous years.

PbS is a very significant direct band gab binary semi-conducting substantial with refractive huge excitations Bohr-radius of 18 nm. It is a very imperative characteristic for the uses of electromagnetic discovery. The energy band gap of wholesale (bulk) crystal and poly-crystalline courses obtained lead Sulfide thin film is approximately 0.35 to 0.5 eV at room temperatures^14^. Therefore the photosensitive constant such as refractive indexes and extinction coefficients of a thin film is very significant for optical sensor uses. In recent times, the sizes altered Lead Sulfide Nanoparticles (grain) are equipped that is reducing the sizes it produces the band-gap of the Lead Sulfide thin films rises. This bounces the quantum confinements in Lead Sulfide Nanoparticles. These characteristics make the quantum confinement effect more prominent in Lead Sulfide associated with other Pb chalcogenide, even for comparatively greater particle size^15,16^.

In this perspective, Lead Sulfide has been developed in numerous procedures by several methods. These are electro-depositions, Spray pyrolysis, and Photo accelerated biochemical depositions^17^, void evaporations solid–gas depositions^18^. Generally, the provision of thin films had been applied through several procedures such as D-C magnetrons spluttering^19^, pulses electro-deposition techniques^20^, thermal vaporization technique^21^ rf magnetrons sputtering^22^, Sprays Pyrolysis technique^23^ as well as CBD (chemical bath deposition) techniques^24^. When a chemical bath is equated with the above approaches, it is comparatively simpler, more quick and cheap. Moreover, through this technique, thin films can be prepared on a glass substrate regardless of the form and surface morphology of the substrate, approximately at 37 °C. Depending on these causes, chemical bath deposition techniques provide, as it is found to be extremely gorgeous by researchers pointing to gain thin film^25^.

The chemical solution method is a thin film placing approach onto substrates as solutions; it includes sources of metallic, hydroxides and sulfides^26^. Chemical bath deposition has become a gorgeous technique based on many reasons, comprising ease of engineering, inexpensive, appropriateness for huge measure deposition area and the capability to deposit a thin film on several substrates and ease of monitoring thin film’s characteristics by adjusting the deposition constraints^27^.

The highest benefit of this technique is that throughout grows of thin films onto a substrate, and it is conceivable to guarantee that the reactions happening in the depositions bath occurred adequately gently^27^. Thin films synthesized by this method have promising claims in a solar cell absorber as well as solar controls coverings windows varnishing uses in earnest weathers, etc.^28,29^. The biological production of thin-film is attainment care of investigators due to the green (biosynthesis) has no influence on the environment. In the present study, Avocado (*Glycosmis cochinchinensis*) Leaf extract was used. Because in the entire world we only used the fruit of Avocado (*Glycosmis cochinchinensis*) Leaf extract and deposed its leaf in the environment and this leaves because the pollution of water, air and land; and this pollution causes the greenhouse effect and acid rain. So that researchers are much emphasized organizing the Nano-sized Lead Sulfide thin film from Avocado (*Glycosmis cochinchinensis*) Leaf extracts by using CBD technique.

The varieties of avocado cultivars in Ethiopia are around six kinds recorded for fabrication which are fusions of original competitions. These include Hass, Fuerte, Pinkerton, Nabal, Bacon, as well Ettinger^30^. But in a large area growing ordinary one which has large leafs harvest of fruits with big seeds. The improvement listed above has big fruit and small seed with small leaves. In this study, the researchers choose the conservative or unimproved avocado variety. In the current research, a methodical study was conducted on PbS thin films grown from CBD technique. The thin film was analyzed by XRD, UV–Vis, scanning-electron-microscopy and Photoluminescence spectrometer to investigate the structural, Morphological and Optical characteristics.

## Materials and methods

### Preparation PbS thin films

PbS thin film was prepared on metal substrates (30 × 70 × 1) mm through CBD techniques.

**Figure 1:**
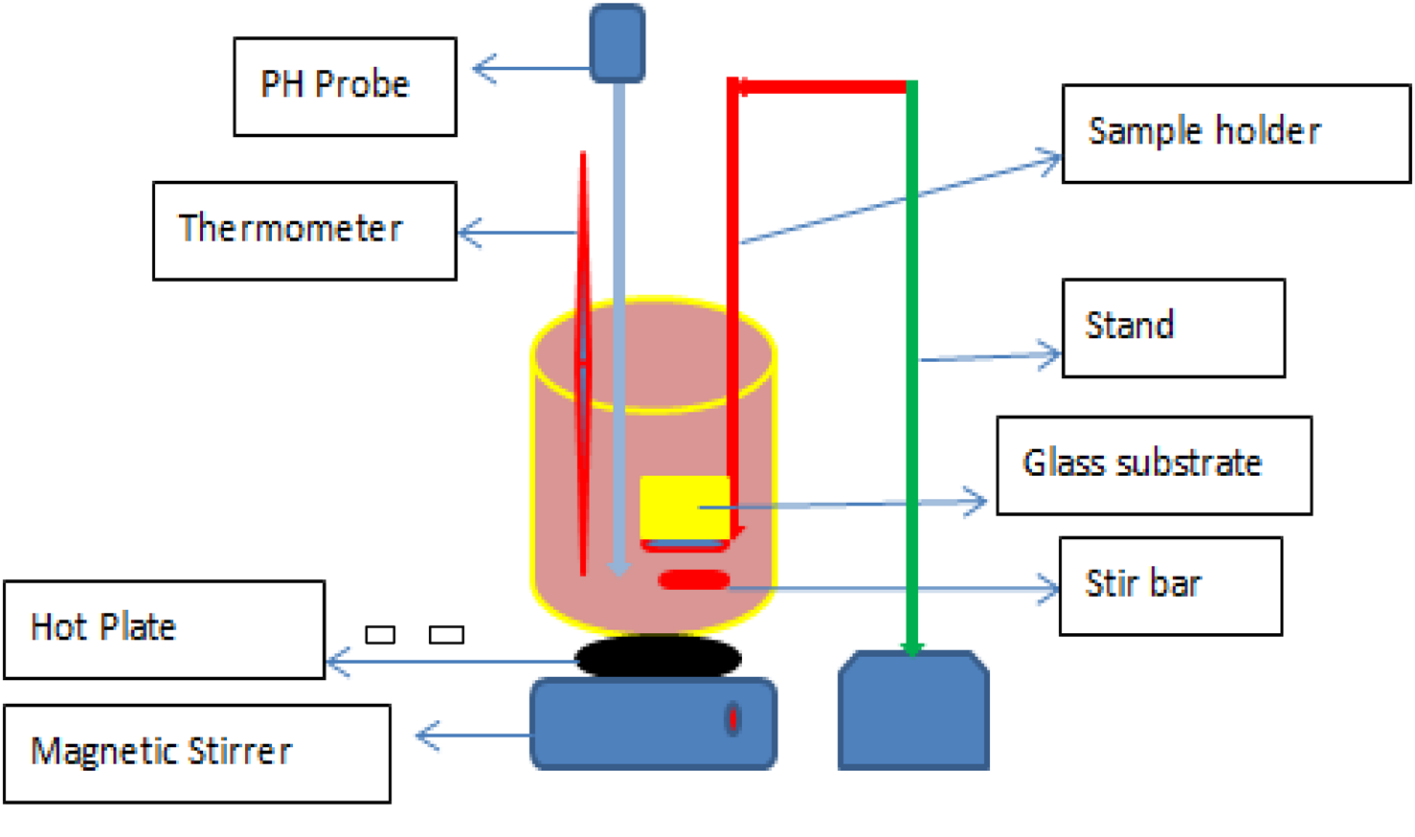
A schematic set of CBD method.

**Figure 2:**
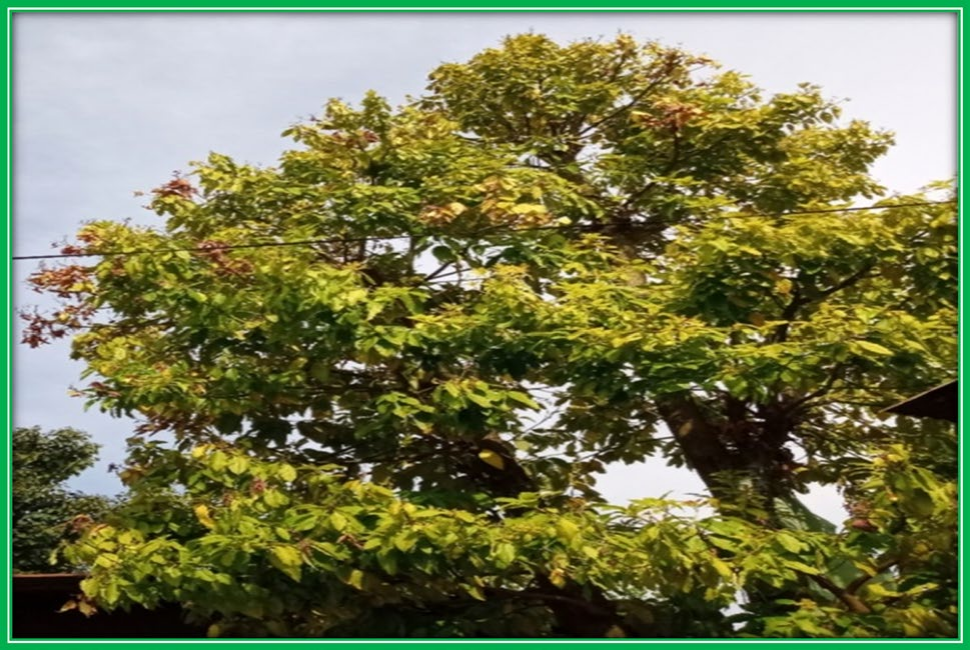
Avocado (*Glycosmis cochinchinensis*) Leaf extracts plant and its fruit taken from local area of Dambi Dollo Town, Oromia, Ethiopia.

An aqueous solution of lead nitrite served as metal precursors basis that is lead(Pb), sodium-sulfide used as sulfides ion sources and [(HOC_2_H_4_)3N] as a reagent for preparations of PbS thin film. Every compound was logically arranged before the fabrications and bath solution was prepared with distilled water. Stepwise, 10 mL of (0.2) Molarity of Pb(NO_3_)_2_ was mixed with 15 mL of Tri-ethanol-amine. Then, 15 mL of (0.2) Molarity of Sodium Sulfide (Na_2_S) were added drop by drop to the solutions^30–32^. pH value of subsequent solutions was well-ordered through droplets of sulfuric acids than with repeated stirrings. The washed metallic substrates were purchased from the workshop to produce Nanoparticle of PbS, with changing pH values as 2, 4, 6 and 8 interleaved in ethanol nearby 15 min, followed through ultra-sonically splashed in distilled water again for 15 min, and finally dried very well. Figure 1 shows the setup of chemical bath deposition techniques. Avocado (*Glycosmis cochinchinensis*) plant used in this study is illustrated in Fig. 2.

### Lead sulfide (PbS) synthesis with Avocado (*Glycosmis cochinchinensis*) Leaf extraction

20 g of Avocado (*Glycosmis cochinchinensis*) Leaf extract was collected and dried under normal conditions for two weeks. Then by using mortar and pestle, it was grinded, and Avocado (*Glycosmis cochinchinensis*) Leaf extract powder was gained. 20 g of powder was added to solutions of PbS and continuously stirred with a magnetic stirrer for 120 min total deposition time. Finally, we bring the water from the chemical solutions through the usage of syringes, and the bottom left molten (gel) of Lead Sulfide is Nanofluids. Succeeding, the glass substrate was glazed with liquefied Lead Sulfide, and it was dehydrated in warm air, wallowed with distilled water and reserved in an oven for supplementary investigation. The plant we have used in this report was cultivated in the local area of Dambi Dollo Town, Oromia, Ethiopia. This study complies with relevant international, national, institutional and legislative guidelines.

The reaction process can be considered as the following steps^23^.

1 $${\text{Pb}}\left( {{\text{NO}}_{{3}} } \right)_{{2}} \left( {{\text{aq}}} \right) \, + {\text{ Na}}_{{2}} {\text{S }}\left( {{\text{aq}}} \right) \to {\text{2NaNO}}_{{3}} \left( {{\text{aq}}} \right) + {\text{ PbS}}\left( {\text{s}} \right)$$

23

4 $${\text{S}}^{{{2} - }} \left( {{\text{aq}}} \right) \, + {\text{ Pb}}^{{{2} + }} \left( {{\text{aq}}} \right) \to {\text{PbS}}\left( {\text{s}} \right)$$

### Characterization techniques

**Figure 3:**
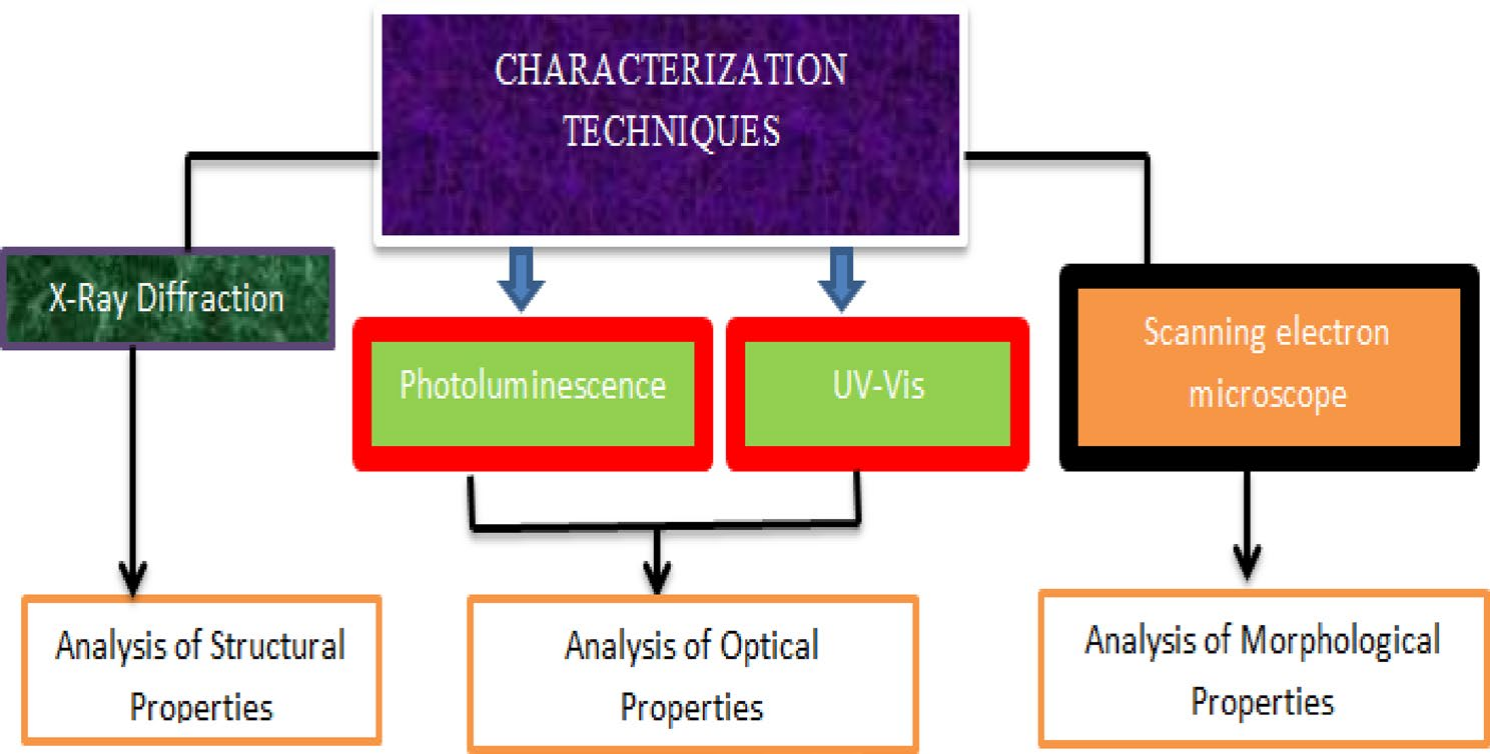
Characterization techniques and its uses.

The structures, morphology and optical characterizations of Lead Sulfide (PbS) were studied through XRD, SEM, and UV–vis spectrophotometer (UV) and PL. Figure 3 shows the characterization techniques with benefits.

## Results and discussion

### Structure characteristics of lead sulfide thin films

**Figure 4:**
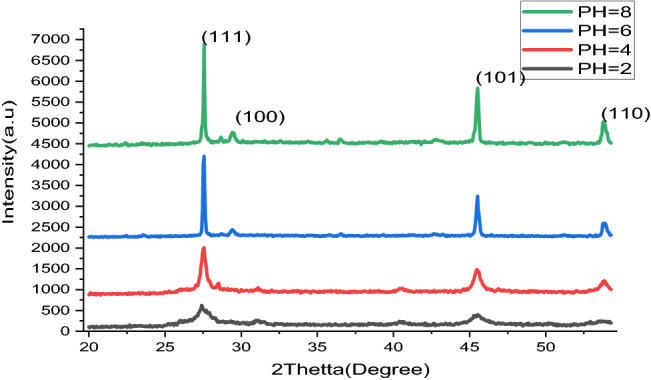
X-Ray Diffraction pattern of Lead Sulfide films from Avocado (*Glycosmis cochinchinensis*) Leaf extracts at different pH values varied as 2, 4, 6 and 8.

Figure 4 revealed the XRD configuration gained from the Lead Sulfide thin film. The detected crests were indexed with a cubic shock salt kind configuration, as inveterate through a standard (JCPDS N0. 05-0592) as well as they are epitomized by their consistent indices in the continuums. The constricted crests display that the materials have respectable crystal nature with very tougher specially oriented along the planes (100), (111), (101) and (110), which strength is predominantly liable on the preparation times. Furthermore, the nonappearance of extra peaks conforming to metal clusters and impurity tells virtuous qualities of the thin film. An average crystal size (0.5 nm) was calculated through Debye-Scherer’s formulas^12,33^.

5 $$\mathrm{D }=\frac{K\lambda }{\beta Cos\theta }$$

**Figure 5:**
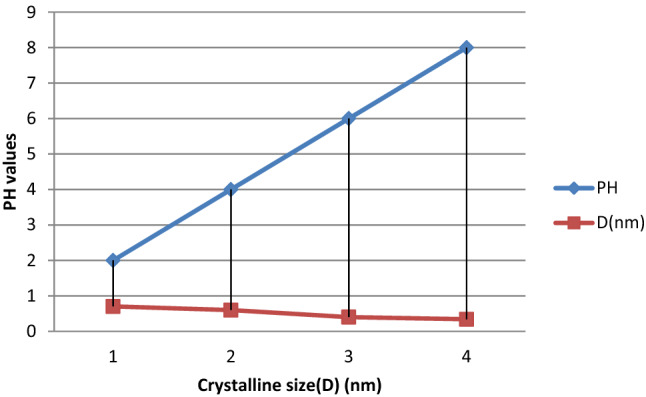
The relationship between crystal size and pH values.

As K is number approximately (0.94), $\lambda$ is the wavelength (0.15418 nm), as well as $\beta$ is the full width at half-maximum of a well-defined deflection peak. The crystal size designates the Nanocrystalline of thin films. As shown in Fig. 4. The XRD revealed that the prepared material is cubic crystal oriented as (111), (110), (100) and (101) crystal planes. This result is in good agreement with reports^34^. Intensification of the energy band gap of the semi-conductor ingredients as the grain size declines; is an influence of quantum imprisonment. This is because of the localization of electrons and hole in a limited interplanetary, inducing visible quantization of the energy. An idea behindhand imprisonment is all almost protection electron, and hole stuck in a small areas^35^. The PbS thin films prepared on metal substrates nucleation’s ratios were measured to be greater than grown rate because of the abundant numbers of nucleation centred that departure on the superficial of the substrates^36^. Table 1 and Fig. 5. Shows the parameters calculated from Scherer’s equation and XRD data. It confirms that crystalline sizes decreased when pH values increased. These reveal the significant influence of pH values on the characteristics of thin films prepared through CBD techniques^37^.

**Table 1:** Parameters of crystal calculated from XRD data.

PH	2θ (°)	θ (°)	FWHM (radian)	D (nm)
2	0.08675	0.43375	247.4849	0.703841
4	0.18234	0.09115	290.8474	0.598906
6	0.2659	0.13295	166.5163	0.402963
8	0.2932	0.2547	178.3113	0.34323

From XRD data, the crystal parameters were determined and discussed in Table 1.

### Photoluminescence spectrum of nano sized lead sulfide (PbS) thin films

**Figure 6:**
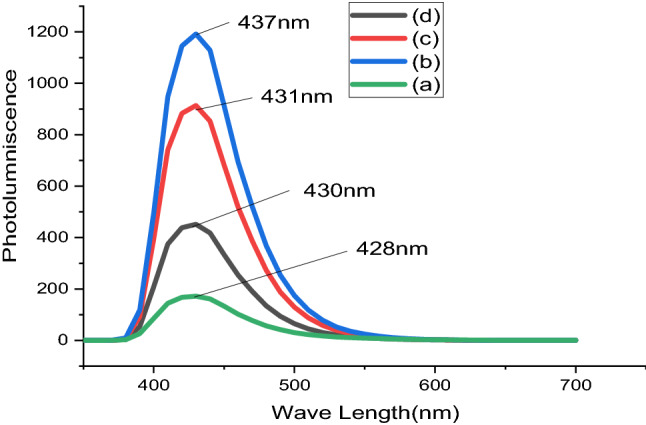
Photoluminescence spectral of PbS thin films from Avocado (*Glycosmis cochinchiinensis*) Leaf extracts at different pH values varied as (**a**) 2, (**b**) 4, (**c**) 6 and (**d**) 8.

Photo-luminescence (PL) spectroscopy is a contactless, non-destructive technique to examine the microelectronic structure of a material. The ghostly dispersal of Photo-luminescence (PL) spectroscopy from a semi-conductor can be studied to non-destructively decide the electrical band gap. This delivers earnings to enumerate the elemental prearrangement of a compound semiconductor and tremendously significant substantial parameters manipulating solar cells expedient effectiveness. The photo-luminescence (PL) spectroscopy continuum at low sample PH values often reveals shadowlike peaks related to contaminations limited with crowd materials. The extraordinary compassion of this method delivers the potential to categorize tremendously little concentration of deliberate as well as accidental impurities that powerfully influence material’s excellence and device performance. The amount of photo-luminescence (PL) spectroscopy produced from materials is in a straight line associated with the comparative quantity of radiate and nonradioactive recombination’s duties. Non-radiate rate is characteristically related to impurities, and therefore, this method can qualitatively screen alterations in substantial excellence as a purpose of growing and dispensation circumstances. Figure 6 displays that the photo-luminescence continuum of Lead Sulfide thin-film dropped at dissimilar PH values varied as (a) 2, (b) 4, (c) 6 and (d) 8. The PbS thin film deposited at PH = 4 shows the highest spectrum; this may be because acidity influences the photoluminescence spectrum of materials^38,39^.

### Superficial morphology characteristics

**Figure 7:**
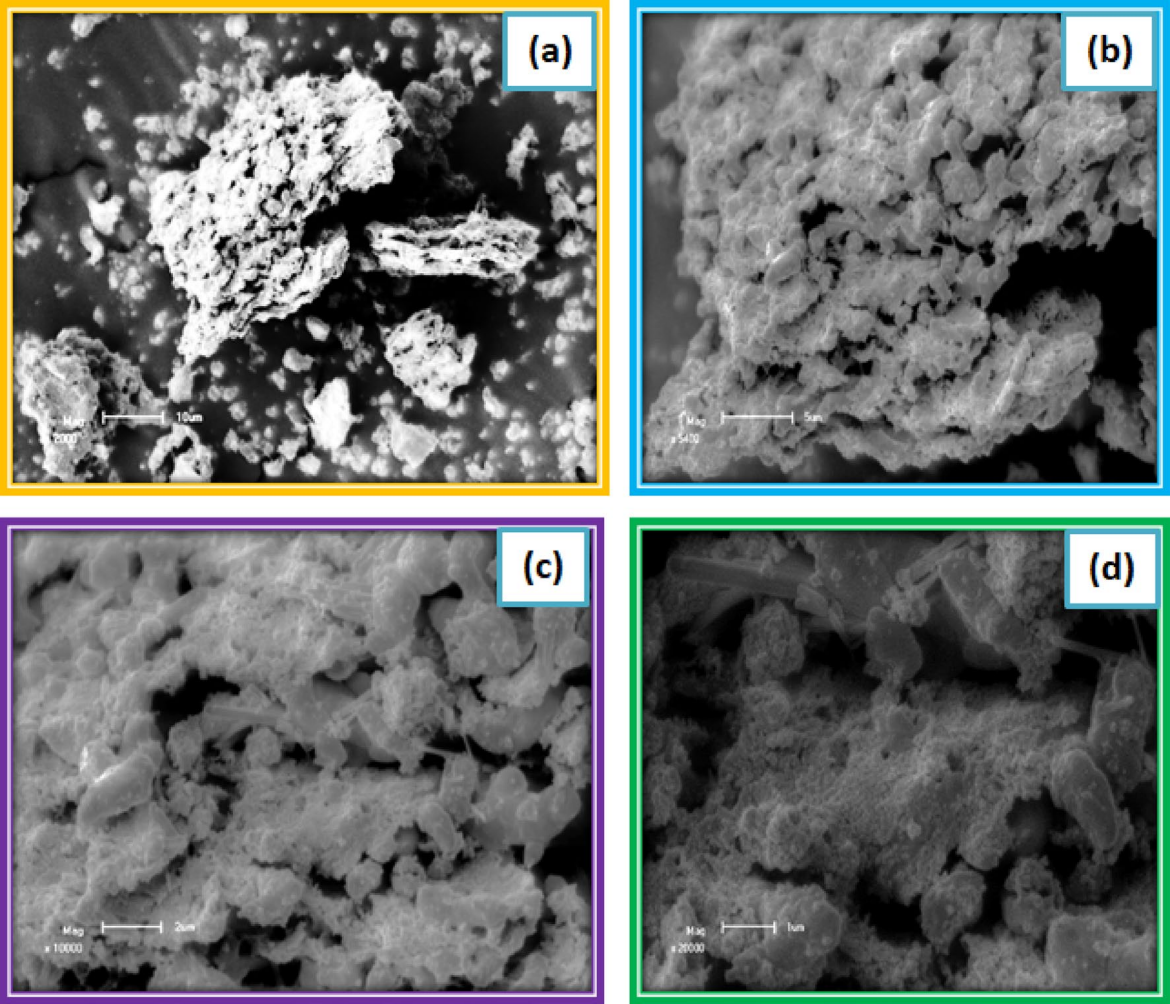
Scanning electron microscopic image of Lead Sulfide thin films from Avocado (*Glycosmis cochinchinensis*) Leaf extract at different PH values varied as (**a**) 2, (**b**) 4, (**c**) 6 and (**d**) 8.

The morphologies of the Nanocrystalline PbS thin film were premeditated through scanning electron micrographs, as perceived in Fig. 7. Clarification of the films in footings of sizes and morphologies of Nanocrystalline Lead Sulfide equipped through CBD technique. Enormously even and supporter Lead Sulfide films are established at pH values 4, 6 and 8. It is noticed that the films are smooth, homogeneous, brilliantly sheltered to the substrates and exhibitions separate groups of Lead Sulfide thin-film except a film prepared at pH = 2, which shows some uneven cracks^14^. The surfaces of the prepared Lead Sulfide film layers display groups in moderately movable thick configuration. This can explain why Lead Sulfide thin film synthesized on substrates is dense and by trivial crystalline sizes of particle^15^.

### Optical properties analysis of nano sized PbS nanoparticles

**Figure 8:**
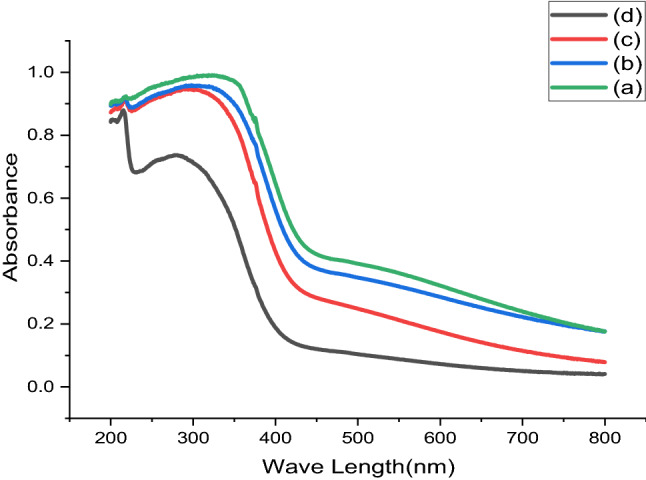
UV–Vis spectral of Lead Sulfide thin films from Avocado (*Glycosmis cochinchinensis*) Leaf extract at different pH values varied as (**a**) 2, (**b**) 4, (**c**) 6 and (**d**) 8.

**Figure 9:**
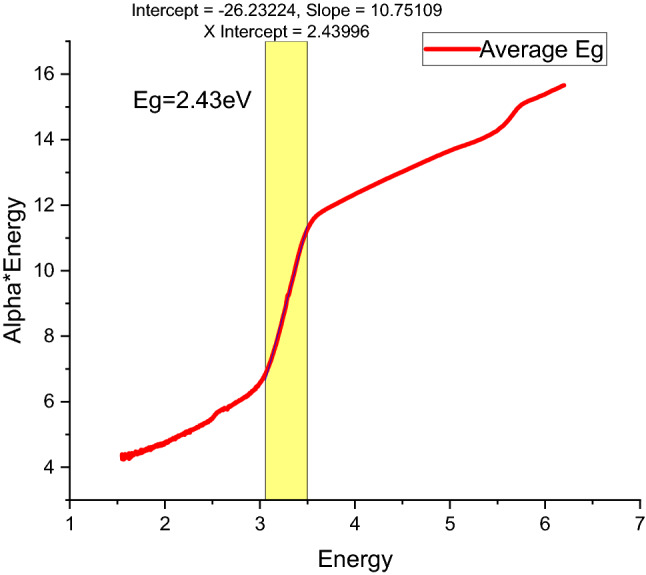
Average energy band-gap of Lead Sulfide film calculated by Tau relation.

In the current study, the researchers studied the influence of the confession constraints on the photosensitivity characteristics of green approach nano-crystallite Lead Sulfide thin films. This work establishes significant means of outlining energy band configurations of semi-conductors. These include transmission as well as absorbance spectrum and the band gaps of Lead Sulfide thin films^16^. Figures 8 and 9 display absorbance and average energy band gap of Lead Sulfide thin films deposited at changed pH values varied as (a) 2, (b) 4, (c) 6 and (d) 8. From all samples at pH = 2 highest spectrum, it reveals that acidic solution influences the optical characteristics of the constituents^17^. An average energy gap of Nanocrystalline PbS thin films is calculated by Tau relation^18^, found to be 2.43 eV which is greater than bulk because of confinements of Nano Crystalline Lead Sulfide thin films, and PL also confirms this result. The obtained result clearly agreed with reports^40^.

### Thicknesses of PbS thin films

**Figure 10:**
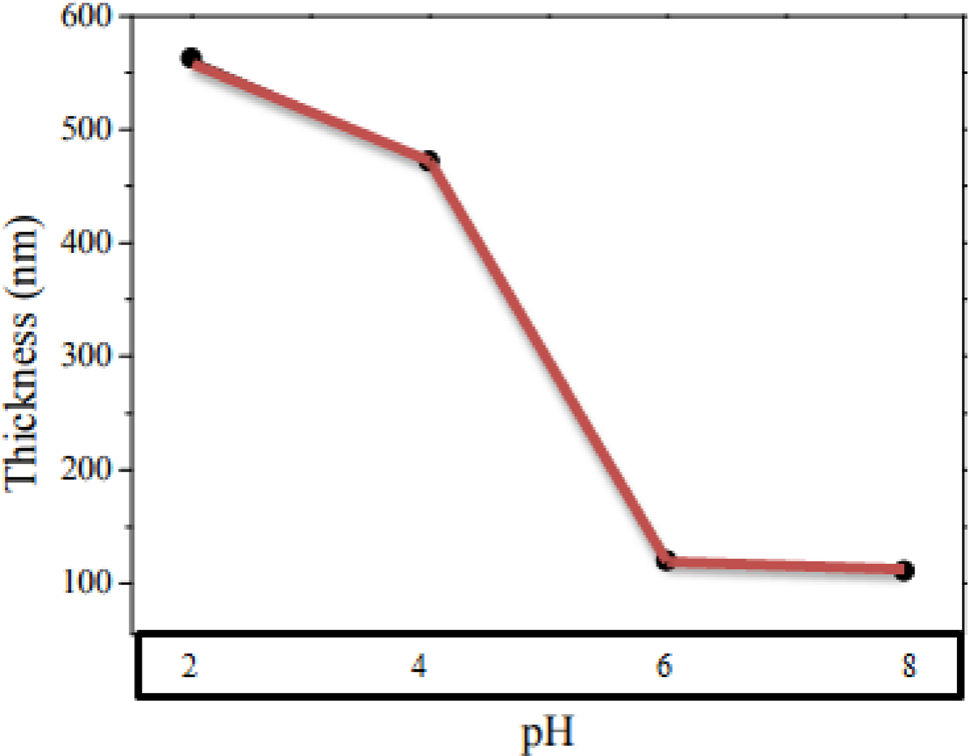
Thicknesses of PbS thin films prepared at changed pH value for a constant deposition time of 2 h.

Thicknesses of the Lead Sulfide thin film deposited was indomitable by heaviness variance technique^41,42^. The densities of the PbS is engaged at 7.6 g/cm^3^ in the wholesale arrangement. Figure 10 displays the difference of the thicknesses for PbS film as a utility of pH values of the combination of the reaction for a constant depositions times of 2 h. It is observed that from Fig. 10, the thicknesses of the prepared thin film reduced from 563 to 111 nm with a rise in pH value. The pH value can be efficiently used to regulate the rate of PbS creations.

## Conclusion

In the current work, Nanocrystalline Lead Sulfide thin films were successfully prepared through the CBD technique from leaf extracts of the Avocado (Glycosmis Cochinchinensis) plant. The produced thin film was of reputable superiority. In preparation of Lead Sulfide, thin films on metal substrates are attained by engaging Lead Nitrate, Sodium Sulfide, and tri-ethanol-amine as sources of precursors and complexing agents, respectively. XRD revealed that the prepared material is cubic crystal oriented as (111), (110), (100) and (101) and crystal plane vertical way to the planes of the substrates and average crystalline size was 0.5 nm. The great absorbance and little reflectance characteristics make fine material for solar cells. The band gap of the Nanocrystalline Lead Sulfide thin film is 2.43 eV which is greater than the bulk because of quantum confinements of Lead Sulfide Nano Crystalline. Photo-luminescence spectroscopy displays the emission at 355–490 nm. Scanning electron micrograph investigation had shown the establishment of cubic structured particles with uniform sizes of supplies. The thicknesses of the prepared thin films were reduced from 563 to 111 nm with increasing pH value. The samples prepared at pH = 4 show good performance, and thin films deposited from Avocado (*Glycosmis cochinchinensis*) Leaf extracts are a promising method to empower pollution remediation and future energy.

## Data Availability

The data are included with in the article.

## References

[1] Batool M, Shafeeq A, Haider B, Ahmad NM (2021). TiO_2_ nanoparticle filler-based mixed-matrix PES/CA nanofiltration membranes for enhanced desalination. Membranes.

[2] Khoo YS, Lau WJ, Liang YY, Karaman M, Gürsoy M, Ismail AF (2021). Eco-friendly surface modification approach to develop thin film nanocomposite membrane with improved desalination and antifouling properties. J. Adv. Res..

[3] Shrestha B, Ezazi M, Kwon G (2021). Engineered nanoparticles with decoupled photocatalysis and wettability for membrane-based desalination and separation of oil-saline water mixtures. Nanomaterials.

[4] Ma G, Almansoori Z, Khorshidi B, Sadrzadeh M (2021). Development of antifouling thin film nanocomposite polyamide membrane using ITO nanoparticles. J. Mater. Sci. Eng.

[5] Ghaffar A, Kiran S, Rafique MA, Iqbal S, Nosheen S, Hou Y, Afzal G, Bashir M, Aimun U (2021). Citrus paradisi fruit peel extract mediated green synthesis of copper nanoparticles for remediation of Disperse Yellow 125 dye. Desalin. Water Treat..

[6] Vinayagam R, Pai S, Varadavenkatesan T, Pugazhendhi A, Selvaraj R (2021). Characterization and photocatalytic activity of ZnO nanoflowers synthesized using Bridelia retusa leaf extract. Appl. Nanosci..

[7] Ngom I, Ndiaye NM, Fall A, Bakayoko M, Ngom BD, Maaza M (2020). On the use of *Moringa oleifera* leaves extract for the biosynthesis of NiO and ZnO nanoparticles. MRS Adv..

[8] Li J, Wan F, Guo W, Huang J, Dai Z, Yi L, Wang Y (2020). Influence of α-and γ-Fe_2_O_3_ nanoparticles on watermelon (*Citrullus lanatus*) physiology and fruit quality. Water Air Soil Pollut..

[9] Klomchit A, Calderin JD, Jaidee W, Watla-Iad K, Brooks S (2021). Napthoquinones from *Neocosmospora* sp.—antibiotic activity against acidovorax citrulli, the causative agent of bacterial fruit blotch in watermelon and melon. J. Fungi..

[10] Bhattacharjee C, Dutta S, Saxena VK (2020). A review on biosorptive removal of dyes and heavy metals from wastewater using watermelon rind as biosorbent. Environ. Adv..

[11] Dejen KD, Zereffa EA, Murthy HA, Merga A (2020). Synthesis of ZnO and ZnO/PVA nano composite using aqueous *Moringa oleifeira* leaf extract template: Antibacterial and electrochemical activities. Rev. Adv. Mater. Sci..

[12] Mosquera-Sánchez LP, Arciniegas-Grijalba PA, Patiño-Portela MC, Guerra-Sierra BE, Munoz-Florez JE, Rodríguez-Páez JE (2020). Antifungal effect of zinc oxide nanoparticles (ZnO-NPs) on *Colletotrichum* sp., causal agent of anthracnose in coffee crops. Biocatal. Agric. Biotechnol..

[13] Bakir, A., Hamimed, S., Landoulsi, A. & Chatti, A. *Biogenic Zinc Oxide Nano-structures Differentiation under Musical Sounds* (2021)

[14] Nurman S, Yulia R, Irmayanti I, Noor E, Candra Sunarti T (2021). Optimizing anti-inflammatory activities of arabica coffee ground (*Coffea arabica* L.) nanoparticle gel. Jundishapur J. Nat. Pharm. Prod..

[15] Pavelkova R, Matouskova P, Hoova J, Porizka J, Marova I (2020). Preparation and characterization of organic UV filters based on combined PHB/liposomes with natural phenolic compounds. J. Biotechnol. X..

[16] Desalegn T, Ravikumar CR, Murthy HA (2021). Eco-friendly synthesis of silver nanostructures using medicinal plant *Vernonia amygdalina* Del. leaf extract for multifunctional applications. Appl. Nanosci..

[17] Song C, Huang M, White JC, Zhang X, Wang W, Sarpong CK, Jamali ZH, Zhang H, Zhao L, Wang Y (2020). Metabolic profile and physiological response of cucumber foliar exposed to engineered MoS_2_ and TiO_2_ nanoparticles. NanoImpact..

[18] Baghaienezhad M, Boroghani M, Anabestani R (2020). Silver nanoparticles synthesis by coffee residues extract and their antibacterial activity. Nanomed. Res. J..

[19] Garrido-Hernández A, Medina-Velazquez DY, Morales-Ramírez ADJ, Molina-Morales M, Barrón-Meza MA, Ramírez-Quirós Y, Reyes-Miranda J (2021). Effect of europium on the blue–green emission of ZnS thin films by polyol and dip-coating technique. Mater. Sci. Semicond. Process..

[20] Borisova ON, Doronkina IG, Feoktistova VM (2021). Resource-saving nanotechnologies in waste water treatment. Nanotechnol. Construct..

[21] Tatarinov DA, Sokolnikova SR, Myslitskaya NA (2021). Applying of chitosan-TiO_2_ nanocomposites for photocatalytic degradation of anthracene and pyrene. J. Biomed. Photon. Eng..

[22] El Shafey AM, Abdel-Latif MK, Abd El-Salam HM (2021). The facile synthesis of poly (acrylate/acrylamide) titanium dioxide nanocomposite for groundwater ammonia removal. Desalin. Water Treat..

[23] Palomino-Merino R (2013). Chemical bath deposition of PbS: Hg^2+^ nanocrystalline thin films. J. Nanomater..

[24] Juliani A, Rahmawati S, Yoneda M (2021). Heavy metal characteristics of wastewater from batik industry in Yogyakarta area, Indonesia. Int. J..

[25] Wibowo E, Rokhmat M, Rahman DY, Murniati R, Abdullah M (2017). Batik wastewater treatment using TiO_2_ nanoparticles coated on the surface of plastic sheet. Procedia Eng..

[26] Pereira, B.M.P. & Backx, B.P. *Nanotechnology in Water Treatment: An Optimistic Perspective for the Near Future*. (2021).

[27] Reyes, K.R. & Robinson, D.B. WO_3_/TiO_2_*Nanotube Photoanodes for Solar Water Splitting with Simultaneous Wastewater Treatment* (Sandia National Laboratories, Springfield, 2013). 10.1021/am403369p 24195676

[28] Banerjee, A., Sarkar, A., Acharya, K. & Chakraborty, N. *Nanotechnology: An Emerging Hope in Crop Improvement* (2021).

[29] Painuli, S., Semwal, P., Bachheti, A., Bachheti, R.K. & Husen, A. Nanomaterials from non-wood forest products and their applications. In *Nanomaterials for Agriculture and Forestry Applications*, 15–40 (Elsevier, 2020).

[30] Devasia J, Muniswamy B, Mishra MK (2020). Investigation of ZnO nanoparticles on in vitro cultures of coffee (*Coffea arabica* L). Int. J. Nanosci. Nanotechnol..

[31] Mokria M, Gebrekirstos A, Said H, Hadgu K, Hagazi N, Dubale W, Bräuning A (2022). Volume estimation models for avocado fruit. PLoS ONE.

[32] Keijok WJ, Pereira RHA, Alvarez LAC, Prado AR, da Silva AR, Ribeiro J, de Oliveira JP, Guimarães MCC (2019). Controlled biosynthesis of gold nanoparticles with *Coffea arabica* using factorial design. Sci. Rep..

[33] García-López JI, Niño-Medina G, Olivares-Sáenz E, Lira-Saldivar RH, Barriga-Castro ED, Vázquez-Alvarado R, Rodríguez-Salinas PA, Zavala-García F (2019). Foliar application of zinc oxide nanoparticles and zinc sulfate boosts the content of bioactive compounds in habanero peppers. Plants..

[34] Bakir, A., Hamimed, S., Landoulsi, A. & Chatti, A. *Biogenic Zinc Oxide Nano-structures Differentiation under Musical Sounds* (2021).

[35] Zhang H, Banfield JF (1999). New kinetic model for the nano-crystalline anatase-to-rutile transformation revealing rate dependence on number of particles. Am. Miner..

[36] Mathpal MC, Tripathi AK, Singh MK, Gairola SP, Pandey SN (2013). Effect of PH value on Raman spectra of Fe: TiO_2_ nanoparticles. Chem. Phys. Lett..

[37] Chung CK, Liao MW, Lai CW (2009). Effects of oxygen flow ratios and PH values on Raman and photoluminescence of titanium oxide thin films deposited by reactive magnetron sputtering. Thin Solid Films.

[38] ChuAbdulrahmanng AF, Ahmed SM, Hamad SM, Barzinjy AA (2021). Effect of growth temperature on morphological, structural, and optical properties of ZnO nanorods using modified chemical bath deposition method. J. Electron. Mater..

[39] Maria KH, Sultana P, Asfia MB (2020). Chemical bath deposition of aluminum doped zinc sulfide thin films using non-toxic complexing agent: Effect of aluminum doping on optical and electrical properties. AIP Adv..

[40] Cohen MM (2014). Tulsi-Ocimum sanctum: A herb for all reasons. J. Ayurveda Integr. Med..

[41] Yoshida K, Chang JF, Chueh CC, Higashihara T (2022). Hybridization of an n-type semi-conducting polymer with PbS quantum dots and their photovoltaic investigation. Polym. J..

[42] Khan ZR, Shkir M (2022). Improved opto-nonlinear and emission properties of spray pyrolysis grown Nd: PbS nanostructured thin films. Phys. B.

